# Use of the Afirma® Gene Expression Classifier for Preoperative Identification of Benign Thyroid Nodules with Indeterminate Fine Needle Aspiration Cytopathology

**DOI:** 10.1371/currents.eogt.e557cbb5c7e4f66568ce582a373057e7

**Published:** 2013-02-11

**Authors:** Syed Z. Ali, Stephanie A. Fish, Richard Lanman, Gregory W. Randolph, Julie Ann Sosa

**Affiliations:** Professor of Pathology, Johns Hopkins University School of Medicine; Memorial Sloan Kettering Cancer Institute and Associate Professor of Medicine (Endocrinology) Weill Cornell Medical College; Chief Medical Officer, Veracyte, Inc.; Associate Professor of Surgery (Otology and Laryngology), Harvard Medical School; Professor of Surgery (Surgical Oncology), Duke University School of Medicine

## Abstract

Ruling out malignancy in thyroid nodules historically depended on thyroid resection and histopathological evaluation until fine needle aspiration (FNA) biopsy was introduced into the United States in the 1970's. Thyroid FNA biopsy identified a majority of thyroid nodules as benign, obviating the need for surgery in over half of the patients. However, 15%-30% of thyroid FNAs have indeterminate cytology that still requires operation, even though most of these operated nodules prove to be benign post-operatively. In order to predict which cytologically indeterminate thyroid nodules are benign and to potentially avoid surgery on these nodules, a recently described commercially available Gene Expression Classifier (GEC) test (Afirma®, Veracyte, Inc., South San Francisco, CA) has been developed that can be run on the FNA sample. This paper reviews the published literature and technology assessments/guidelines by independent parties and professional groups regarding the clinical utility as well as the analytic and clinical validity of the Afirma GEC.

## Clinical Scenario

Thyroid nodules are common and typically benign. However, given that 5-10% of nodules are malignant, current practice guidelines recommend evaluation with ultrasound followed by fine needle aspiration (FNA) biopsy for most clinically significant thyroid nodules.^1^^,^ 2 ^,^ 3 Most diagnostic FNA biopsies are read as cytopathologically benign or malignant, but 15%-30% remain indeterminate.^2^ Most patients with indeterminate lesions (defined in the Bethesda System as Atypia of Undetermined Significance or Follicular Lesion of Undetermined Significance, suspicious for Follicular or Hürthle Cell Neoplasm and suspicious for malignancy) are referred for a diagnostic thyroid surgery.^4^ Approximately three-quarters of these nodules are ultimately found to be benign on final surgical pathology.^5^^,^ 6

In 2011, it is estimated that more than 450,000 thyroid FNAs were performed. In that same year, approximately 48,020 primary thyroid malignancies were diagnosed.^7^ In order to avoid diagnostic surgery on benign thyroid nodules with indeterminate FNA cytopathology, pre-operative FNA-based genomics tests should predict a risk of malignancy comparable to the risk of malignancy in a cytologically benign nodule that is resected (“approximately 5% or less”).^8^ At this level of risk, physicians can confidently recommend clinical and sonographic monitoring *in lieu* of thyroid resection as they do for cytologically benign nodules under current clinical management schemas.^9^ Recent reviews have evaluated known gene mutation marker panels associated with thyroid malignancy and the Afirma GEC towards this end.^10^^,^ 11 ^,^ 12 A recent meta-review of a panel of somatic mutation markers associated with malignancy such as BRAF, RAS, RET/PTC, and PAX8/PPARgamma found sensitivity to be too low (63.7%) to achieve a high enough negative predictive value (NPV) to recommend monitoring when these mutations are absent.^13^ The Afirma GEC employs a different approach analyzing the mRNA expression of 167 genes with high enough sensitivity (92%) in indeterminate cytology lesions to identify the signature of a benign thyroid nodule with 95% NPV: that is, similar to the risk of malignancy in a resected thyroid nodule with a preoperatively benign FNA cytopathology diagnosis.^14^^,^ 15

## Test Description

When needle passes are made for cytologic analysis of sonographically suspicious thyroid nodules, two dedicated passes also are made for Afirma GEC analysis and immediately stored in nucleic acid preservative solution. If the FNA cytopathology is nondiagnostic, benign, or malignant, the sample collected for the Afirma GEC is discarded. If the FNA cytopathology is indeterminate, the Afirma GEC sample undergoes RNA extraction and nucleic acid amplification. Processed Afirma GEC samples are hybridized to a custom Afirma Thyroid microarray and analyzed with a classification algorithm using linear support vector machine logic to produce either a “Benign” or “Suspicious” test result. About 10% of FNA samples have inadequate RNA yield or quality and are reported by the Afirma GEC as “No Result”.^16^

## Public Health Importance

The incidence of thyroid cancer in the U.S. has risen dramatically. In 2009, there were 37,200 new cases of thyroid cancer, while in 2013, it is anticipated there will be 60,220 new cases.^17^^,^ 18 At the same time, there has been an increase in the utilization of thyroid FNA and subsequent thyroid surgery.^19^ The prevalence of thyroid nodules increases with age and is more common in females. Approximately 50% of women ≥50 years have at least one thyroid nodule based on published ultrasound and autopsy series.^20^ Two thirds of thyroid nodules have benign FNA cytopathology and monitoring is implemented, whereas those with indeterminate or malignant cytology are generally referred for surgery. Because thyroid nodules with indeterminate FNA cytopathology have a 25% risk of malignancy when resected, 75% of these operations will likely be on nodules determined to be benign post-operatively.^5^^,^ 6 Thyroid surgery is associated with potential complications, including temporary and permanent hypocalcemia, recurrent laryngeal nerve injury (with voice change, dysphagia, and potentially airway compromise if bilateral), and bleeding, with an incidence as high as 2-10%.^21^^,^ 22 ^,^ 23 While there is strong evidence that high volume thyroid surgeons on average have fewer complications than low volume counterparts, 50% of thyroid operations in the U.S. are still performed by surgeons who perform ≤5 thyroidectomies/year.^24^ Hypothyroidism is an expected sequelae of thyroid surgery, with patients requiring life-long thyroid hormone supplementation or replacement therapy.

## Published Reviews, Recommendations and Guidelines

***Systematic evidence reviews.*** Palmetto Government Benefits Administrators (Palmetto GBA), the CMS Medicare Administrative Contractor with oversight for the Afirma GEC, has published its ****assessment of the test as an update to its local coverage article on molecular diagnostics. This review determined that the test meets criteria for analytical and clinical validity, and clinical utility as a reasonable and necessary Medicare benefit, effective January 1, 2012.^25^

***Recommendations by independent groups.*** As part of the CLIA Laboratory licensure process, the analytical and clinical validation data for the Afirma GEC were independently assessed by reviewers from the California Department of Public Health and the New York State Department of Health.^26^^,^ 27 Both of these reviews resulted in a favorable licensure outcome.

***Guidelines by professional groups.*** The National Comprehensive Cancer Network (NCCN) thyroid carcinoma guidelines were updated in December, 2012 to state “Molecular diagnostics may be useful to allow reclassification of follicular lesions (follicular neoplasm or follicular lesion of undetermined significance) as more likely to be benign or more likely to be malignant…If molecular testing predicts a risk of malignancy comparable to the risk of malignancy seen with a benign FNA cytology (approximately 5% or less), consider observation.” The NCCN guidelines for abnormal gene/gene expression profile testing are associated with Level of Evidence 2A (lower level evidence, uniform NCCN consensus that the intervention is appropriate).^8^^,^ 28

## Evidence Overview

***Analytical Validity*: test accuracy, reliability in measuring differences in expression of relevant genes (analytic sensitivity and specificity), and robustness.**

• Building on an earlier study by Chudova et al.^14^, a large collaborative study by Walsh et al. reviewed over 30 sub-studies on the Afirma GEC, finding high analytic sensitivity and specificity after dilution of thyroid neoplasm FNA samples with adjacent normal tissue or benign neoplasms (such as nodular hyperplasia and lymphocytic thyroiditis), as well as dilution with blood and genomic DNA, respectively.^14^^,^ 29

• High reproducibility was found in studies of interlaboratory concordance (R^2^ 0.98), as well as intra-assay, inter-assay, and intra-nodule concordance (R^2^ 0.99, 0.98, and 0.95, respectively).^29^

• The assay was robust to a wide range of temperature, storage and stressed shipping conditions and was reproducible across different operators, runs, and reagent lots with routine use of control reagents/samples for in-process Quality Control monitoring.^29^

***Clinical Validity*: test accuracy in correctly determining which indeterminate cytology FNA biopsies are benign compared to expert surgical histopathology.**

• Two prospective multicenter studies evaluated the negative predictive value (NPV) for the Afirma GEC, which is the key diagnostic performance metric used to make a decision to monitor patients* in lieu* of referral for diagnostic thyroid surgery.^14^^,^ 15 Both studies utilized diagnosis of the surgical pathology specimen by a central panel of blinded academic endocrine histopathologists as the reference standard for clinical validation. Approximately one quarter of study sites were academic and three quarters were community-based (total sites n=49).

• In both studies, NPV was >94% for cytologically indeterminate thyroid FNAs with atypia/follicular lesions of undetermined significance and follicular/Hürthle cell neoplasm diagnoses, but lower when the cytology was suspicious for malignancy. Overall, the NPV for the Afirma GEC was similar to the NPV for a resected thyroid nodule with benign cytopathology.^5^^,^ 30

• In the second, larger study^15^, although the NPV was 95% for all indeterminate cytology nodules (at the 24% risk of malignancy expected in clinical practice), the NPV was 85% for FNAs with a cytopathology diagnosis suspicious for malignancy. Sensitivity was 92% for indeterminate nodules overall. Clinical specificity for indeterminate nodules rose from 0% for cytopathology alone to 52% with the Afirma GEC, meaning that half of the benign nodules with indeterminate cytopathology could be identified with this pre-operative test.

***Clinical Utility*: net benefit of test in improving health outcomes by allowing recommendation for monitoring instead of a diagnostic surgery on benign thyroid nodules.**

• Duick et al. was a retrospective analysis based on chart review of 368 patients (395 indeterminate thyroid nodules) cared for by 51 physicians at 21 practice locations in 11 states. When compared to historical controls, the relative rate of operation on cytologically indeterminate nodules fell 90%, from 74% historically to 7.6% with Afirma GEC benign results (p < 0.001).^16^ The comparisons between historical controls and operative rates based on the binomial test achieved more than 99% power in detecting a less than 25% change.^31^ With Afirma GEC benign results, in 92.4% of the cases, physicians recommended watchful waiting *in lieu* of a diagnostic thyroid resection. The residual rate of surgery was similar to the historic 9% resection rate on cytologically benign thyroid nodules and may relate to other clinical parameters and issues of patient and physician preference, including issues related to nodule size.^5^

• These results were consistent with a previously conducted web- and mail-based opinion survey of 84 physician practices, with a mean of 89% of physicians reporting that they recommended watchful waiting for patients with cytologically indeterminate FNAs but benign Afirma GEC results.^32^

***Limitations.*** In the clinical validity studies, thyroid FNAs with indeterminate cytology diagnoses that were cytologically suspicious for malignancy did not have sufficiently low NPV to generally recommend monitoring.^10^ Secondly, the Duick et al clinical utility study used historical rather than contemporary controls.^16^ Although historical controls were used, these were appropriately validated based on a meta-review of 11 recent thyroid pathology studies where thyroid nodule evaluation was similar to current community practice.^5^

## Conclusions

The clinical validity studies reviewed would fall into the Level 1 category in the Evaluation of Genomic Applications in Practice and Prevention (EGAPP) hierarchy of data sources and study designs, based on two prospective, multicenter, double blinded cohort studies.^33^ A validated clinical decision rule was based on classification concordance of Afirma GEC benign results with blinded expert surgical pathology benign diagnosis. 11 Sensitivity in most indeterminate FNAs was high enough (92%) to achieve a NPV of 94-95%, which is comparable to a thyroid nodule that is benign on cytopathology but undergoes surgical resection (93-94%).^5^^,^ 30 However, the Afirma GEC NPV for FNAs where the cytopathology was suspicious for malignancy was only 85%. While this lowers the residual risk of malignancy from 62% to 15%, surgical consultation still should be planned in these patients. The test utilized interlaboratory comparisons in a large collaborative study, an EGAPP criterion for Level 1 hierarchy of analytic validity study design.^34^ Improvement in the net health outcome is based on avoidance of surgery in patients with indeterminate thyroid FNA cytology that would have been found to be benign on surgical pathology. Clinical utility of the Afirma GEC was evaluated in clinical practice, outside of the investigational setting, in an opinion survey and a controlled study consistent with EGAPP Level 3 criteria for clinical utility study design. The Afirma GEC potentially can improve the net health outcome by providing an alternative to diagnostic thyroid surgery, and therein the risk of downstream complications of surgery, in patients with benign thyroid nodules but indeterminate FNA cytopathology. In summary, the studies reviewed here regarding clinical and analytic validity, and clinical utility support recommendation for offering patients the alternative of using the Afirma GEC to monitor patients in lieu of thyroid resection when applied in the specific case of thyroid FNAs where there is indeterminate cytopathology: atypia/follicular lesion of undetermined significance, and follicular/Hürthle cell neoplasm.

## Competing interests

Dr. Lanman is an employee of Veracyte, Inc. The other co-authors have no conflict of interest.
